# Unravelling Cancer Immunity: Coagulation.Sig and BIRC2 as Predictive Immunotherapeutic Architects

**DOI:** 10.1111/jcmm.70525

**Published:** 2025-03-30

**Authors:** Ziang Yao, Jun Fan, Yucheng Bai, Jiakai He, Xiang Zhang, Renquan Zhang, Lei Xue

**Affiliations:** ^1^ Department of Traditional Chinese Medicine Peking University People's Hospital Beijing China; ^2^ Department of Thoracic Surgery The First Affiliated Hospital of Nanjing Medical University Nanjing China; ^3^ Department of Thoracic Surgery First Affiliated Hospital, Anhui Medical University Hefei China; ^4^ Department of Respiratory and Critical Care Medicine The Affiliated Huai'an Hospital of Xuzhou Medical University, the Second People's Hospital of Huai'an Huai'an Jiangsu China

**Keywords:** BIRC2, coagulation, immune checkpoint inhibitor, immunotherapy, pan‐cancer

## Abstract

Immune checkpoint inhibitors (ICIs) represent a groundbreaking advancement in cancer therapy, substantially improving patient survival rates. Our comprehensive research reveals a significant positive correlation between coagulation scores and immune‐related gene expression across 30 diverse cancer types. Notably, tumours exhibiting high coagulation scores demonstrated enhanced infiltration of cytotoxic immune cells, including CD8^+^ T cells, natural killer (NK) cells, and macrophages. Leveraging the TCGA pan‐cancer database, we developed the Coagulation.Sig model, a sophisticated predictive framework utilising a coagulation‐related genes (CRGs) to forecast immunotherapy outcomes. Through rigorous analysis of ten ICI‐treated cohorts, we identified and validated seven critical CRGs: BIRC2, HMGB1, STAT2, IFNAR1, BID, SPATA2, IL33 and IFNG, which form the foundation of our predictive model. Functional analyses revealed that low‐risk tumours characterised by higher immune cell populations, particularly CD8^+^ T cells, demonstrated superior ICI responses. These tumours also exhibited increased mutation rates, elevated neoantigen loads, and greater TCR/BCR diversity. Conversely, high‐risk tumours displayed pronounced intratumor heterogeneity (ITH) and elevated NRF2 pathway activity, mechanisms strongly associated with immune evasion. Experimental validation highlighted BIRC2 as a promising therapeutic target. Targeted BIRC2 knockdown, when combined with anti‐PD‐1 therapy, significantly suppressed tumour growth, enhanced CD8^+^ T cell infiltration, and amplified IFN‐γ and TNF‐α secretion in tumour models. Our findings position the Coagulation.Sig model as a novel, comprehensive approach to personalised cancer treatment, with BIRC2 emerging as both a predictive biomarker and a potential therapeutic intervention point.

## 1 Introduction

The advent of immune checkpoint inhibitors (ICIs) has transformed the landscape of cancer therapeutics, demonstrating remarkable improvements in patient outcomes [1, 2, 3, 4, 5]. Recent epidemiological data from the United States reveal a dramatic surge in ICI utilisation among cancer patients, escalating from 1.54% to 43.63% between 2011 and 2018 [6, 7]. Despite this widespread adoption, therapeutic success remains constrained, with clinical responses observed in merely 12.46% of treated individuals [6]. The therapeutic efficacy exhibits substantial heterogeneity across different malignancies and patient cohorts [8]. Although investigators have elucidated several potential predictive biomarkers, including tumour mutation burden (TMB), tumour‐infiltrating lymphocytes (TILs), distinctive gut oncomicrobiome patterns, and Immunoscore, their prognostic capabilities have shown notable limitations [9, 10, 11, 12, 13]. The lack of a universally applicable biomarker that consistently predicts the efficacy of immune checkpoint inhibitors across different cancer types hinders efforts to enhance patient selection and formulate effective combination therapy strategies.

The tumour coagulome, a network of molecules driving thrombosis or bleeding, is a key focus in cancer research [14]. The coagulome interacts with TME to promote or restrict tumour growth and impacts immune responses [15]. Coagulation activation induces inflammation, recruiting immune cells like macrophages [16]. Anticoagulants can boost antitumor immunity and work as effective ICI adjuvants. Studies suggest DOACs like rivaroxaban enhance ICI effectiveness in melanoma patients [17]. This highlights coagulation's role in TME and its link to tumour immune evasion. We used bioinformatics and predictive modelling to study coagulation's link to TME. The prognostic value of coagulation‐related genes (Coagulation.Sig) in immunotherapy was also studied.

This study developed Coagulation.Sig, validated using transcriptomic data from 10,154 patients across 30 cancers and 10 ICI‐treated cohorts, including 921 individuals from 5 cancer types. Our findings revealed that Coagulation.Sig has the potential to predict ICI outcomes with greater accuracy than previously established signatures across multiple cancer types. In vivo experimental validation demonstrated that targeted suppression of HMGB1 expression, a crucial mediator within the Coagulation.Sig pathway, effectively attenuated the aggressive phenotype of pancreatic cancer cells. These findings provide compelling evidence for HMGB1's fundamental contribution to malignant tumour progression.

## 2 Methods

### 2.1 Pan‐Cancer Transcriptomic Data

To investigate the relationships between the coagulation signature (Coagulation.Sig) and immunosuppression across various malignancies, we utilised multi‐omics datasets using TCGA Pan‐cancer datasets from UCSC Xena (https://xenabrowser.net). The expression data for the TCGA mRNA for the pan‐cancer cohort were normalised for batch effects [18]. In this study, we deliberately excluded three cancer types—acute myeloid leukaemia (LAML), diffuse large B‐cell lymphoma (DLBC), and thymoma (THYM)—due to their predominant composition of immune cells, which could potentially bias our analysis [19].

### 2.2 ICI RNA‐Seq Cohorts

To confirm Coagulation.Sig*'s* prognostic value, we collected transcriptional data and clinical details from ten ICI RNA‐Seq cohorts. This included five datasets for cutaneous melanoma (SKCM)—Liu [20], Hugo [21], Gide [22], Van Allen [23], and Riaz [24]; two compilations for urothelial carcinoma (UC)—Synder [25], Mariathasan [26]; one dataset for glioblastoma multiforme (GBM)—Zhao [27]; one aggregation for gastric cancer (GC)—Kim [28]; and one collection for renal cell carcinoma (RCC)—Braun [29]. Immunotherapies targeting PD‐1, CTLA‐4, PD‐L1, or their combinations were used, with six using anti‐PD‐1, two anti‐PD‐L1, one anti‐CTLA‐4, and one combining anti‐PD‐(L)1 and anti‐CTLA‐4. Specifically, the Hugo [21] dataset included 27 pre‐treatment tumour specimens from 26 individuals, while the Zhao [27] compilation contained 34 pre‐treatment tumour biopsies from 17 subjects. For both datasets, a single tumour specimen was randomly selected per participant to ensure representativeness. To mitigate confounding batch effects arising from different ICI RNA‐Seq datasets, we applied the ComBat‐seq method [30], thereby standardising the data for unbiased comparative analyses.

### 2.3 Clinical Outcomes

Treatment efficacy evaluation encompassed two primary endpoints: Overall Survival (OS) and Objective Response Rate (ORR), with response assessment conducted according to RECIST 1.1 criteria across immunotherapy cohorts. For the Hugo 2016 population, response evaluation adhered to irRECIST guidelines. Patient stratification into response categories was based on therapeutic outcomes: subjects achieving Complete Response (CR) or Partial Response (PR) were classified as responders, whereas those exhibiting Stable Disease (SD) or Progressive Disease (PD) constituted the non‐responder group.

### 2.4 Creation of a Predictive Signature for the ICI Dataset

Integration of five high‐volume RNA‐Seq datasets yielded a consolidated analytical cohort (*n* = 772) comprising patients receiving immune checkpoint inhibitor therapy. This population encompassed three distinct malignancies: RCC (*n* = 181; Braun 2020), UC (*n* = 348; derived from Mariathasan 2018 and Snyder 2017), and SKCM (*n* = 243; sourced from Liu 2019, Gide 2019, and Riaz 2017). The study population underwent stratified allocation, with 80% assigned to model development (*n* = 618) and 20% to initial validation (*n* = 154). A separate testing cohort (*n* = 149) was established through the amalgamation of five additional RNA‐Seq datasets.

### 2.5 Model Training and Hyperparameter Optimisation

Marker gene identification within the tumour coagulome was accomplished through ABESS algorithmic analysis of the training dataset [31]. The development of Coagulation.Sig incorporated six established machine learning approaches: random forest (RF), support vector machine (SVM), k‐nearest neighbours (KNN), Naive Bayes (NB), extreme gradient boosting (XGBoost), and AdaBoost Classification Trees (AdaBoost). Model optimization employed 10‐fold cross‐validation methodology coupled with grid search techniques for hyperparameter refinement, ensuring optimal algorithmic performance. Based on comprehensive AUC (Area Under the Receiver Operating Characteristic Curve) evaluation, we identified the random forest (RF) algorithm as the optimal model, with refined parameters of mtry = 2, ntree = 500, and nPerm = 1, which demonstrated superior predictive accuracy and robustness in distinguishing tumour coagulome marker genes.

### 2.6 Validation of the Model and Independent Testing

We created six distinct models from the training dataset employing various machine learning algorithms. The model exhibiting the highest performance was designated as the definitive Coagulation.Sig for the training set. To assess its predictive efficacy, this selected model was subsequently applied to both the validation and testing datasets.

### 2.7 Comparison of *CoagulationSig* With Other Predictive Gene Signatures

Comparative analysis encompassed six cancer‐associated signatures (INFG.Sig, T.cell.inflamed.Sig, PDL1.Sig, LRRC15.CAF.Sig, NLRP3.Sig, and Cytotoxic.Sig [32, 33, 34, 35, 36]) alongside seven melanoma‐specific molecular signatures (CRMA.Sig, IMPRES.Sig, IPRES.Sig, TcellExc.Sig, ImmunCells.Sig, IMS.Sig, TRS.Sig [37, 38, 39, 40, 41, 42, 43]). Evaluation of pan‐cancer signatures against Coagulation.Sig assessed their respective capacities to predict ICI therapeutic outcomes. For melanoma‐specific analyses, signature performance was evaluated using melanoma cases from the testing cohort, specifically focusing on the Hugo 2016 and Van Allen 2015 populations. Methodological approaches maintained fidelity to original signature protocols: implementing ssGSEA for NLRP3.Sig analysis, cancer‐class methodology for ImmunCells.Sig evaluation, and expression profiling for TcellExc.Sig assessment. Cytotoxic.Sig quantification utilised the geometric mean of PRF1 and GZMA expression values [36].

### 2.8 Functional Annotation

SsGSEA and GSEA were performed using the MSigDB, along with the GSVA [44] and clusterprofiler [45] R packages.

### 2.9 Assessment of Immune Infiltration Using CIBERSORT

CIBERSORT uses gene expression deconvolution and support vector regression to estimate cell abundances in tumour microenvironments [46]. It identifies 22 immune cell subsets through normalised transcriptomic data analysis. Immune cell infiltration data from CIBERSORT were obtained from Torsson et al.'s pan‐cancer immune landscape study [47].

### 2.10 Comprehensive Immune Profiling From Danaher et al.

Immune infiltration scores were obtained from Danaher et al.'s analysis of the TCGA pan‐cancer dataset [48]. Immune cell populations were quantified using 60 marker genes. This approach identified 14 immune cell types, including TILs, CD8^+^ T cells, Tregs, DCs, B cells, NK cells, and mast cells. These evaluations were validated for robustness and reliability through consistent analyses.

### 2.11 Immune Signature Evaluation

Twenty‐nine immune signatures were identified based on He et al.'s research. The GSVA R package and ssGSEA method were used to evaluate signature enrichment across samples [44].

### 2.12 Immunogenomic Indicator Calculation

Immunogenomic indicators were obtained from Torsson et al.'s pan‐cancer analysis [47]. Quantification of intratumoral heterogeneity (ITH) employed subclonal genomic fraction analysis, where subclonal components represent non‐predominant tumour populations as defined by the ABSOLUTE algorithm. This computational framework delineates tumour‐specific alterations as composite mixtures of clonal and subclonal elements, each characterised by distinct ploidy states. Copy number profiles were parameterized using two key metrics: n_segs, denoting the total segment count within sample profiles, and frac_altered, representing the proportion of genomic bases deviating from baseline ploidy. Aneuploidy quantification incorporated cumulative scoring of chromosomal arm‐level amplifications and deletions. Immune receptor diversity assessment encompassed both T‐cell receptor (TCR) and B‐cell receptor (BCR) repertoires, utilising Shannon entropy and richness indices derived from tumour RNA sequencing data.

### 2.13 Mutational Signature Analysis in Cancer Genomes

The maftools R package performed NMF on mutations categorised by 96 trinucleotide contexts in TCGA samples. The derived mutation signatures were compared with those in the COSMIC database. Cosine similarity metrics assessed the correspondence between the two mutation signature sets.

### 2.14 Gene Set Enrichment in Oncogenic Pathways

Enrichment scores for 10 key oncogenic pathways (totaling 187 oncogenes) were derived from Sanchez‐Vega et al.'s research [49]. Pathway enrichment scores were calculated using ssGSEA with the GSVA R package [44].

### 2.15 Cell Culture

The LLC‐1 and A549 cell lines were obtained from the American Type Culture Collection (ATCC). Both cell types were cultured in RPMI 1640 medium with 10% fetal bovine serum (FBS) (Gibco, Grand Island, NY, USA) and 1% penicillin/streptomycin solution under conditions of 37°C in a 5% CO_2_ atmosphere.

### 2.16 Establishment of a Stable Cell Line via Lentiviral Transduction

To establish a stable cell line, lentiviral transduction was performed. Cells were infected with lentivirus containing the target gene, followed by selection with puromycin to ensure the integration and stable expression of the target gene in the cell line. Target sequence: GCCGAATTGTCTTTGGTGCTT (Ref Seq: NM_001166) for A549 cell line, GCAGGACATTCTACTCTCTTT (Ref Seq: NM_007465) for LLC‐1 cell lines.

### 2.17 CCK‐8 Assay for Cell Viability

Cells were plated at 3000 cells per well in a 96‐well plate (MedChemExpress, Cat. No: HY‐K0301). CCK‐8 reagent was added at 0, 24, 48, and 72 h, followed by a 2‐h incubation at 37°C. Absorbance at 450 nm was measured using a microplate reader to determine cell viability.

### 2.18 Colony Formation

We inoculated 500 cells into each well of a 6‐well plate, permitting independent growth to facilitate the formation of separate colonies. After a period of 14 days, the cells were fixed using methanol, followed by staining with crystal violet to visualise the colonies. Subsequently, images of the stained colonies were captured, and the total number of colonies formed was counted.

### 2.19 Flow Cytometry for Apoptosis Detection

Cells were collected, washed, and stained with 5 μL Annexin V and propidium iodide (PI) per sample, then incubated in the dark at room temperature for 15 min. After staining, the cell suspension was immediately analysed using a flow cytometer. Data was collected through the flow cytometer, and cell viability, early apoptosis and late apoptosis/necrosis were differentiated based on the staining patterns of Annexin V and PI, allowing for quantitative analysis of apoptosis levels in the cell population.

### 2.20 Establishment of a Subcutaneous Tumour Model

LLC shNC and shBIRC2 tumour cells were prepared in sterile PBS for subcutaneous tumour model creation in 6‐ to 8‐week‐old female C57BL/6 mice. Mice were anaesthetised, and 1 × 10^6^ cells in 100 μL of sterile PBS were injected subcutaneously into each mouse's back. We regularly monitored tumour growth, measuring tumour size with callipers every 2–3 days to evaluate tumour volume. Once the tumour reached a predetermined size, we proceeded with further experiments or treatments. This model is commonly used in cancer research to study tumour biology and test anti‐cancer treatments.

### 2.21 Preparation of Mouse Tumour Single‐Cell Suspension

After excising the mouse tumour tissue, we washed it in sterile PBS to remove blood and impurities. We cut the tumour tissue into small pieces (about 1–2 mm^3^) to facilitate subsequent digestion. We placed the tissue fragments in a digestion enzyme solution containing collagenase I/IV and DNase, and incubated at 37°C with gentle shaking for 60 min until a single‐cell suspension was obtained. The digested suspension was filtered through a 70 μm strainer to eliminate tissue fragments, washed with PBS, and centrifuged to remove enzymes. We treated it with red blood cell lysis buffer for 5 min to remove erythrocytes, resuspended it in cold PBS, and counted cells to adjust the concentration. The prepared single‐cell suspension was then ready for flow cytometry analysis.

### 2.22 Flow Cytometry for Analysing the Tumour Immune Microenvironment in Mice

We began the procedure by employing the Zombie NIR Fixable Viability Kit (Biolegend, Cat. No: 423106) and the Zombie Violet Fixable Viability Kit (Biolegend, Cat. No: 423114) to remove dead cells from our analysis. Subsequently, the cells were incubated for 30 min at 4°C with anti‐mouse αCD45 (Biolegend, Cat. No: 103132; 157,214), αCD3 (Biolegend, Cat. No: 100204), αCD4 (Biolegend, Cat. No: 100422), and αCD8a (Biolegend, Cat. No: 100751) antibodies at the recommended concentrations. For intracellular cytokine staining of IFN‐γ (Biolegend, Cat. No: 505830) and TNF‐α (Biolegend, Cat. No: 506306) in T cells, the cells were fixed and permeabilised after a 5‐h stimulation at 37°C in a 5% CO_2_ atmosphere. This stimulation was achieved by the addition of Monensin sodium salt (Abcam, Cat. No: ab120499, 1 μg/mL), Ionomycin calcium salt (PeproTech, Cat. No: 5608212, 100 ng/mL), and Phorbol 12‐myristate 13‐acetate (PMA, Abcam, Cat. No: ab120297, 100 ng/mL).

### 2.23 Statistical Analysis

Statistical computations and graphical representations were implemented in R (v4.3.1). Continuous variable comparisons employed the Wilcoxon rank‐sum test, while relationship assessments utilised Spearman's correlation coefficient. ICI therapeutic efficacy evaluation incorporated receiver operating characteristic (ROC) curve analysis, executed via the pROC package. Optimal threshold determination derived from ROC curve analysis of the training cohort, utilising clinical response as the primary endpoint. Survival analyses were conducted through Kaplan–Meier methodology using the survival package, with statistical significance established at *p* < 0.05 for all two‐sided comparative analyses.

## 3 Results

### 3.1 Analysing Coagulation Scores and Immune Activation in the Pan‐Cancer TCGA Cohort

**FIGURE 1:**
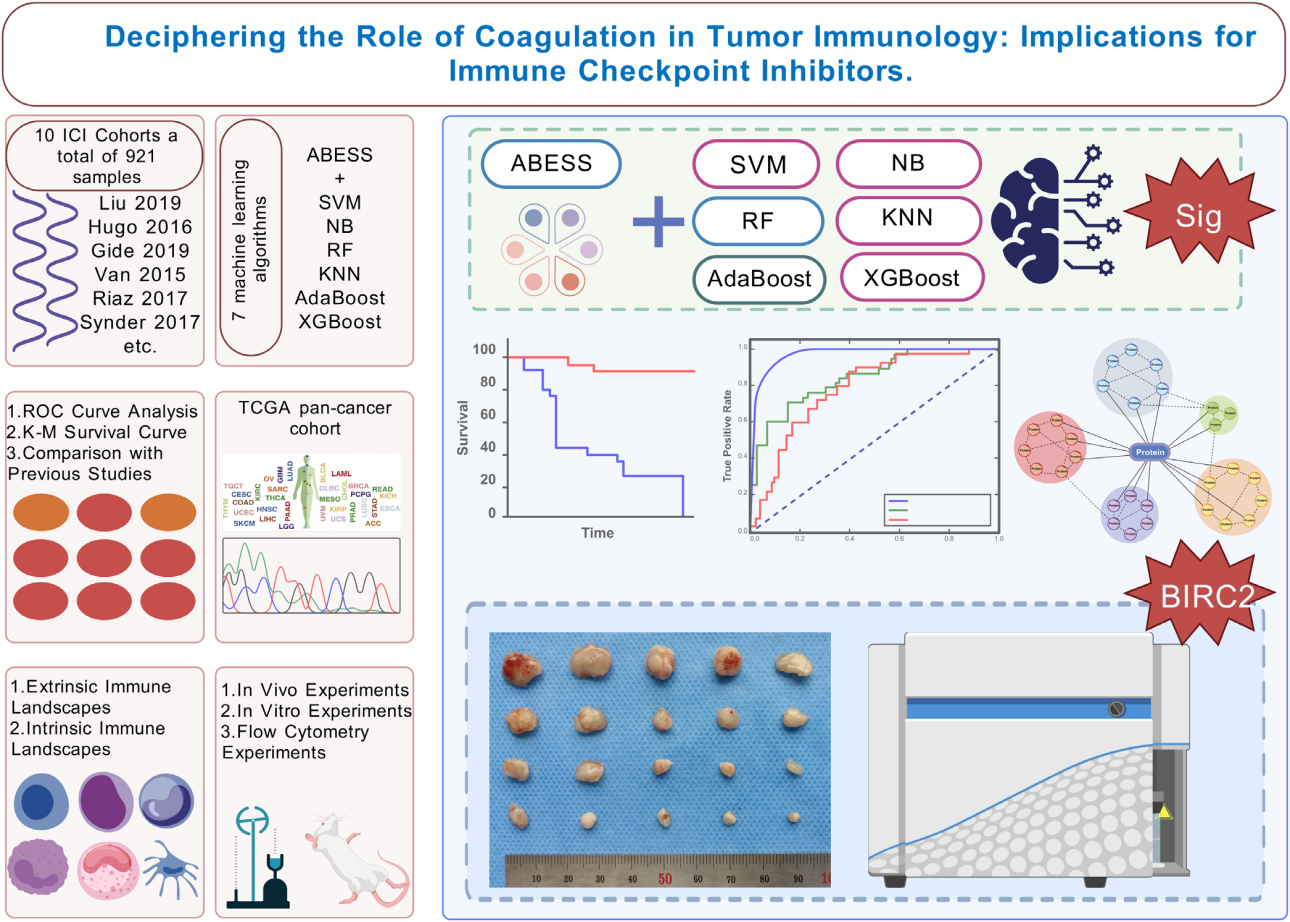
An illustration of the general workflow adopted in this study.

**FIGURE 2:**
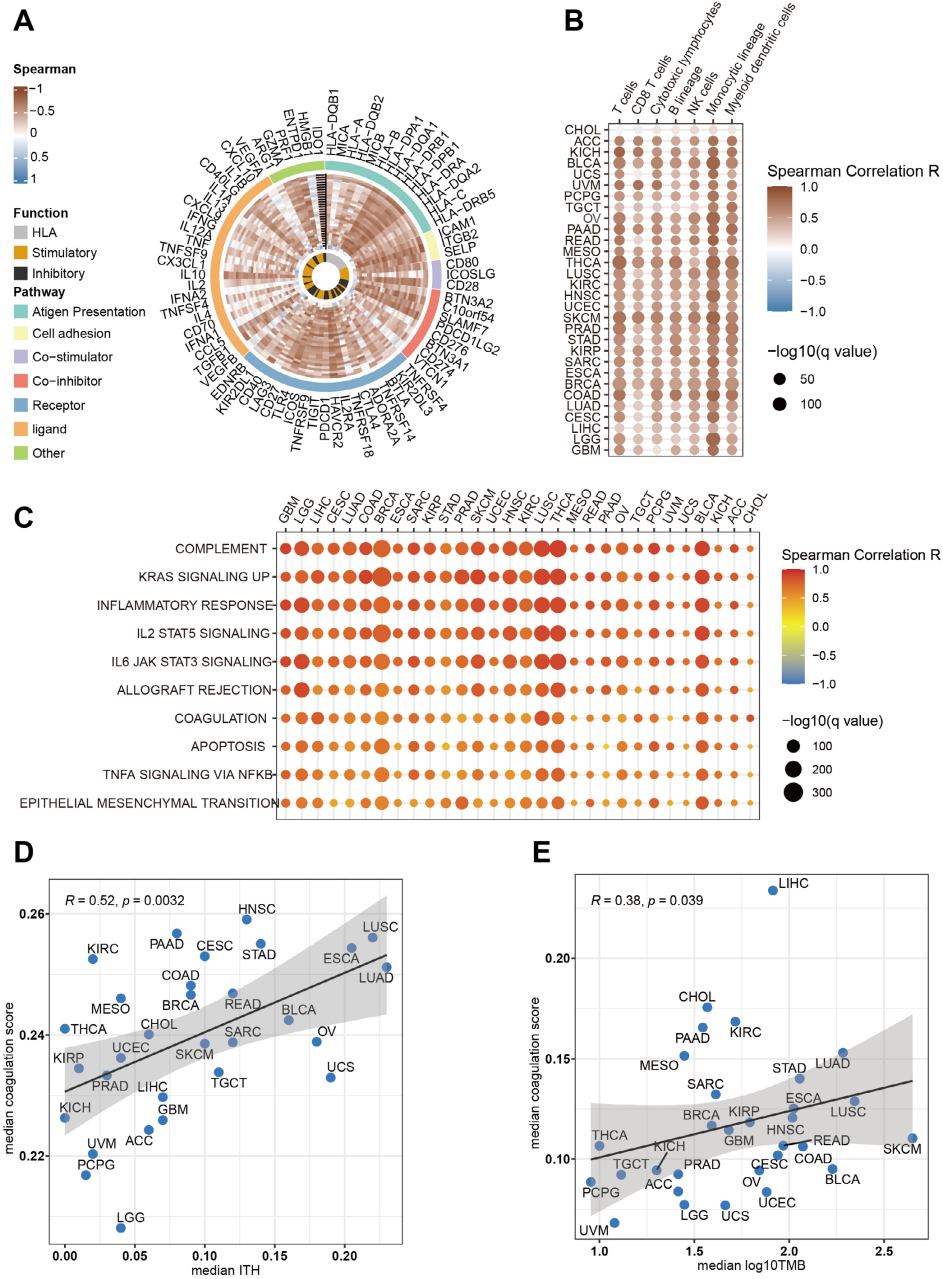
Comprehensive analysis of associations between Coagulation.Sig and immune infiltration, along with tumour characteristics, across multiple cancer types in the TCGA cohort. (A) The Circos plot illustrates correlations between Coagulation Signature (Coagulation.Sig) activity, represented by GSVA scores, and immune‐related gene expression across various cancers. The colour gradient reflects Spearman correlation values, ranging from −1 to 1, with genes categorised into immune functions such as antigen presentation, HLA, stimulatory, inhibitory and other immune pathways. (B) This heatmap displays the relationship between Coagulation.Sig activity and infiltration levels of immune cells, including T cells, B cells, and macrophages, across different cancer types. Dot size indicates statistical significance (−log10(*q* value)), and colour represents the Spearman correlation coefficient. (C) A bubble heatmap highlights the association between Coagulation.Sig activity and key immune pathways, such as interferon gamma response, IL6 JAK STAT3 signalling, and inflammatory response, across cancer types. Dot size denotes statistical significance (−log10(*q* value)), and colour shows the correlation strength (Spearman *R*). (D) Scatter plot illustrating the correlation between median Coagulation.Sig activity scores and median tumour mutational burden (TMB) across various cancers. Each point represents a specific cancer type, with a shaded area indicating the confidence interval around the regression line. (E) Scatter plot depicting the relationship between median Coagulation.Sig activity scores and median intratumor heterogeneity (ITH) across different cancer types. Similar to (D), Spearman correlation coefficient (*R*) and *p*‐values are provided for each plot.

To elucidate the complex interactions between coagulation and immunity, we developed a comprehensive analytical framework (Figure 1). GVSA assessed coagulation scores in the TCGA pan‐cancer cohort, showing a strong positive correlation with immune‐related gene expression across 30 cancer types (Figure 2A). To better understand the TME, we analysed immune cell infiltration, finding that higher coagulation scores correlated with increased cytotoxic cells, including CD8^+^ T cells, NK cells, and macrophages (Figure 2B). These findings collectively indicate that heightened coagulation scores are associated with enhanced anti‐tumour immunity. Furthermore, hallmark pathways related to coagulation scores were examined to ascertain whether immunosuppressive functions were diminished in tumours characterised by high coagulation scores. Significant activation of pathways such as the complement cascade, inflammatory response, and IL2 STAT5 signalling was observed (Figure 2C), all of which are recognised for their roles in facilitating robust immune responses [50, 51, 52]. A positive correlation between coagulation scores and ITH was identified (*r* = 0.52, *p* = 0.003, Figure 2D). A similarly positive association was identified between coagulation scores and TMB, which is a recognised factor relevant to immune response (*r* = 0.38, *p* = 0.039, Figure 2E). These results collectively indicate that tumours with elevated coagulation scores display markedly improved anti‐tumour immune responses.

### 3.2 Predicting Immunotherapy Outcomes Using Coagulation.Sig

**FIGURE 3:**
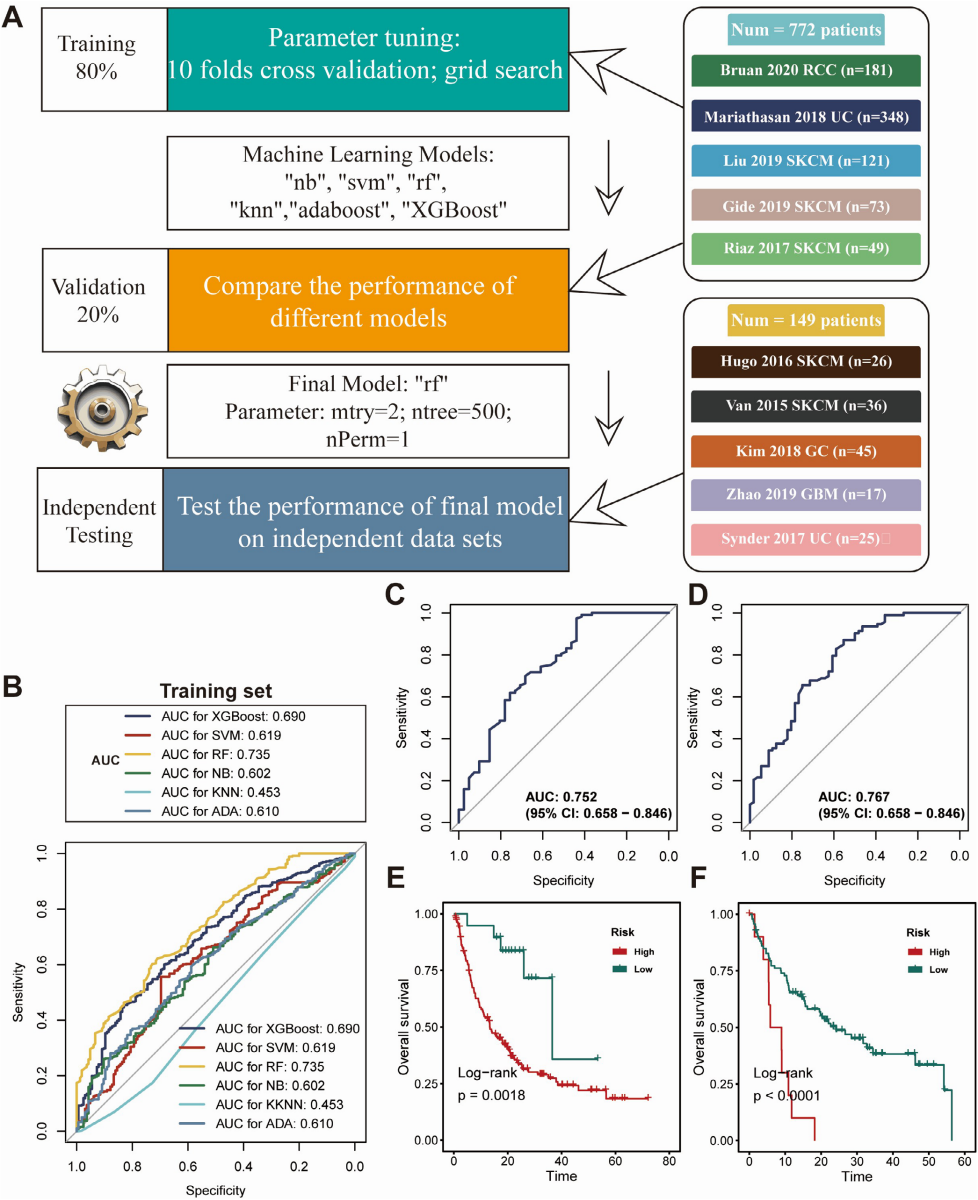
Construction and assessment of a machine learning model for predicting the Coagulation Signature (Coagulation.Sig). (A) Diagram outlining the machine learning approach to establish and validate the Coagulation.Sig model. Data were divided into 80% for training and 20% for validation. Model tuning involved 10‐fold cross‐validation with a grid search across several algorithms, including Naive Bayes (nb), Support Vector Machine (svm), Random Forest (rf), *k*‐Nearest Neighbours (knn), AdaBoost (adaboost), and XGBoost. The final model chosen was Random Forest (rf) with optimised parameters (mtry = 2, ntree = 500, nPerm = 1), which was then evaluated on independent datasets such as Braun 2020 RCC and Kim 2018 GC. (B) Receiver Operating Characteristic (ROC) curves display the performance of different machine learning models on the training set, with Area Under the Curve (AUC) values provided for XGBoost, SVM, RF, KNN, and AdaBoost. The Random Forest model achieved the highest AUC at 0.735. (C) ROC curve showing the performance of the selected Random Forest model on the validation set, achieving an AUC of 0.752 (95% CI: 0.658–0.846). (D) ROC curve demonstrating the performance of the final model on an independent test set, with an AUC of 0.767 (95% CI: 0.658–0.846). (E, F) Kaplan–Meier survival curves showing differences in overall survival (OS) between high‐risk and low‐risk groups according to Coagulation.Sig scores in both validation and test sets. Significant OS differences were found between the groups, with log‐rank test p‐values (*p* = 0.0018 and *p* < 0.0001, respectively).

Recognising coagulation's fundamental role in anti‐tumour immunity modulation, we developed a Coagulation.Sig to enhance immunotherapeutic stratification. The study integrated bulk RNA sequencing and clinical data from 10 ICI cohorts, encompassing patients receiving anti‐PD(L)‐1, anti‐CTLA‐4, or combination therapy. Dataset partitioning established training (*n* = 620), validation (*n* = 154) and testing (*n* = 149) populations. ABESS algorithmic analysis of the training cohort identified eight pivotal coagulation‐related genes (CRGs): HMGB1, STAT2, BIRC2, IFNAR1, BID, SPATA2, IL33 and IFNG. The analytical framework is illustrated in Figure 3A. Model development incorporated six machine learning approaches, with parameter optimization achieved through 10‐fold cross‐validation and grid search methodologies. Performance evaluation within the training cohort revealed RF as the superior algorithm, demonstrating an AUC of 0.735, thus establishing the definitive Coagulation.Sig (Figure 3B). Subsequent validation confirmed robust predictive capacity, achieving AUCs of 0.75 and 0.77 in validation and independent testing cohorts, respectively (Figure 3C,D). Survival analysis stratified ICI‐treated patients into risk categories using training set‐derived thresholds. Kaplan–Meier analyses demonstrated significantly prolonged overall survival in low‐risk populations across both validation and testing datasets (*p* < 0.01; Figure 3E,F).

### 3.3 Comparing Coagulation.Sig With Other Predictive Gene Signatures

**FIGURE 4:**
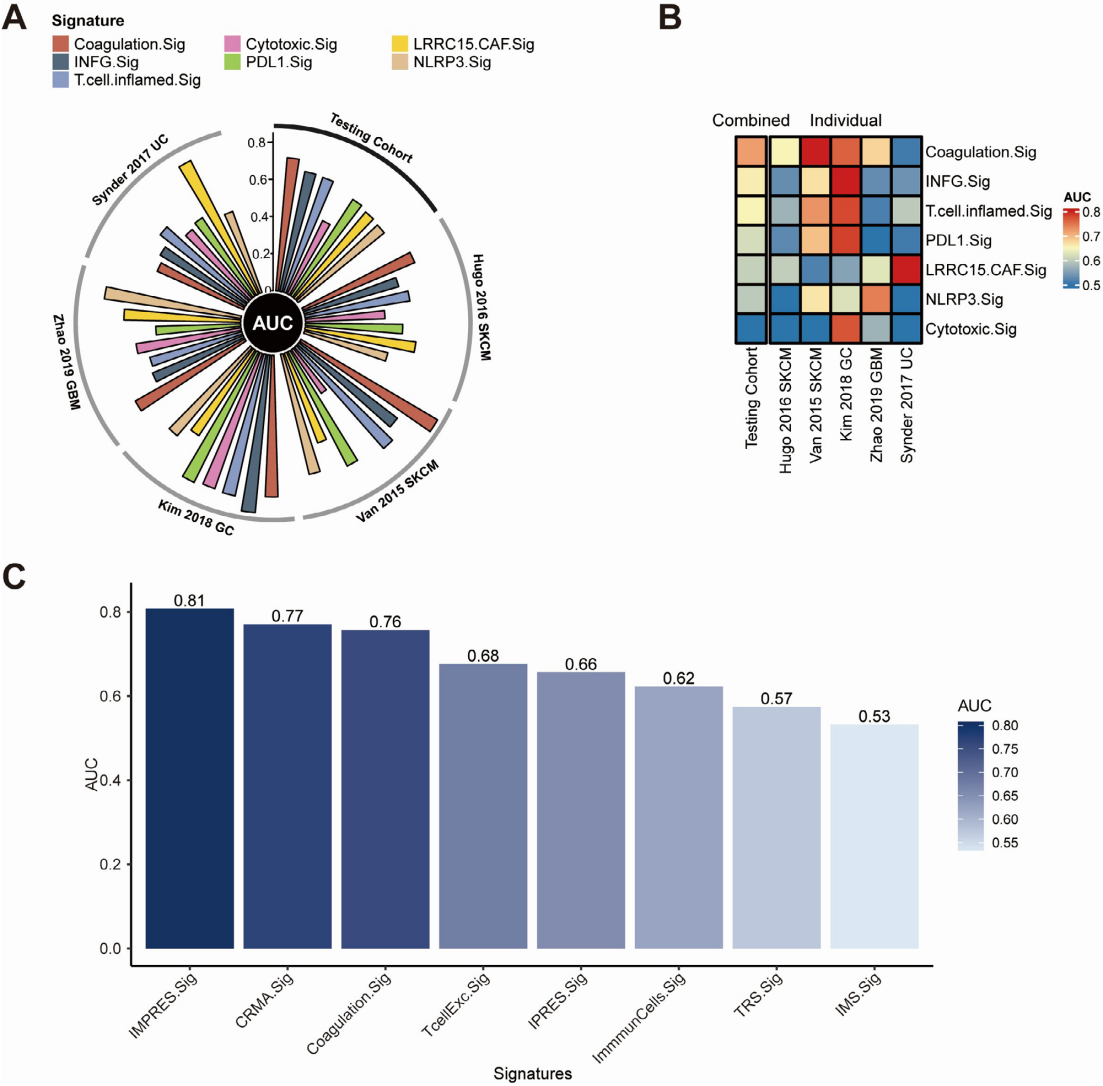
Evaluation of Coagulation Signature (Coagulation.Sig) performance across pan‐cancer cohorts. (A) Circos plot showing Area Under the Curve (AUC) values for various immune‐associated signatures, including Coagulation.Sig, across several validation cohorts such as Snyder 2017 UC, Van 2015 SKCM, Kim 2018 GC, and Zhao 2019 GBM. The radial layout highlights each signature's predictive capability within different datasets. (B) Heatmap depicting AUC scores for both combined and individual coagulation‐related signatures across multiple datasets. The colour scale spans from 0.5 to 0.8, with warmer shades indicating higher predictive performance. (C) Bar chart comparing AUC scores for different immune and coagulation‐related signatures, such as IMPRES.Sig, CRMA.Sig, and Coagulation.Sig, indicating their predictive strengths. Taller bars reflect better AUC performance.

Coagulation.Sig outperformed other ICI‐related signatures in pan‐cancer analysis, achieving an AUC of 0.77 in the testing set, compared to INFG.Sig's AUC of 0.66 (Figure 4A). It showed superior predictive ability across SKCM, UC, and GC, demonstrating versatility for multiple cancer types (Figure 4B). For melanoma‐specific signatures, Coagulation.Sig remained a strong predictor, with an AUC of 0.71 for forecasting ICI response in melanoma patients. However, within this subset, IMPRES.Sig and CRMA.Sig surpassed Coagulation.Sig, achieving slightly higher AUCs of 0.81 and 0.77, respectively (Figure 4C).

### 3.4 Functional Analysis of Coagulation.Sig

**FIGURE 5:**
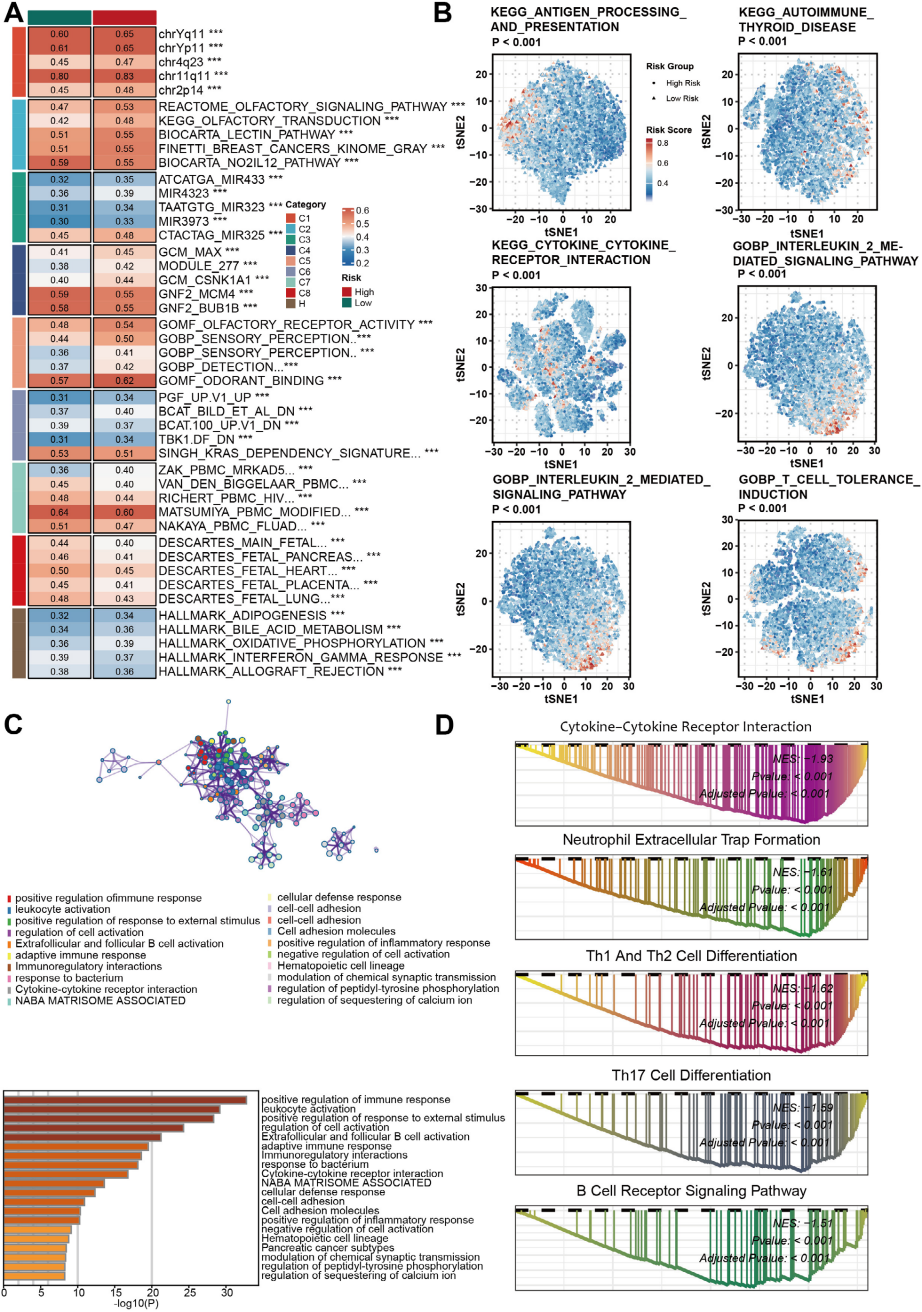
Functional and pathway analysis of the Coagulation Signature (Coagulation.Sig) in Cancer. (A) Heatmap depicting the relationship between gene sets and Coagulation.Sig risk categories across multiple cancer cohorts. Gene modules are divided into functional groups (C1–C8), with risk levels designated as high or low based on Coagulation.Sig scores. Key gene sets highlighted include those related to cell cycle regulation, metabolism and immune function. (B) t‐SNE plots visualising the spread of Coagulation.Sig risk scores across different cancer types, covering pathways like antigen processing and immune response. Risk scores are colour‐coded from low (blue) to high (red), illustrating the link between coagulation activity and significant biological pathways, including immune signalling and inflammatory processes. (C) Upper: Network map showing interactions among gene modules involved in immune response and cell signalling pathways. This network highlights the complex interactions between pathways related to tumour progression and coagulation activity. Lower: Bar plot of the most enriched Gene Ontology (GO) terms associated with immune response, cell activation and inflammation. The length of each bar represents the significance level (−log10(P)) for each GO term, pinpointing critical biological processes linked to coagulation. (D) Gene Set Enrichment Analysis (GSEA) plots for major enriched pathways, such as cytokine‐cytokine receptor interaction, neutrophil extracellular trap formation, and differentiation of Th1, Th2, and Th17 cells. Enrichment scores and *p*‐values are shown, highlighting Coagulation.Sig's involvement in immune cell activation and differentiation processes.

Detailed pathway analysis was conducted to explore Coagulation.Sig's biological mechanisms. Patients in the TCGA cohort were grouped into high‐ and low‐risk categories based on Coagulation.Sig scores, with activated pathways shown in Figure 5A. Immune‐related pathways, such as antigen processing, cytokine interactions, and T cell tolerance, were enriched in the low‐risk group (Figure 5B). Over Representation Analysis (ORA) and GSEA confirmed immune pathway enrichment in the low‐risk group (Figure 5C,D).

### 3.5 Extrinsic Immune Landscapes in High‐ and Low‐Risk Groups

**FIGURE 6:**
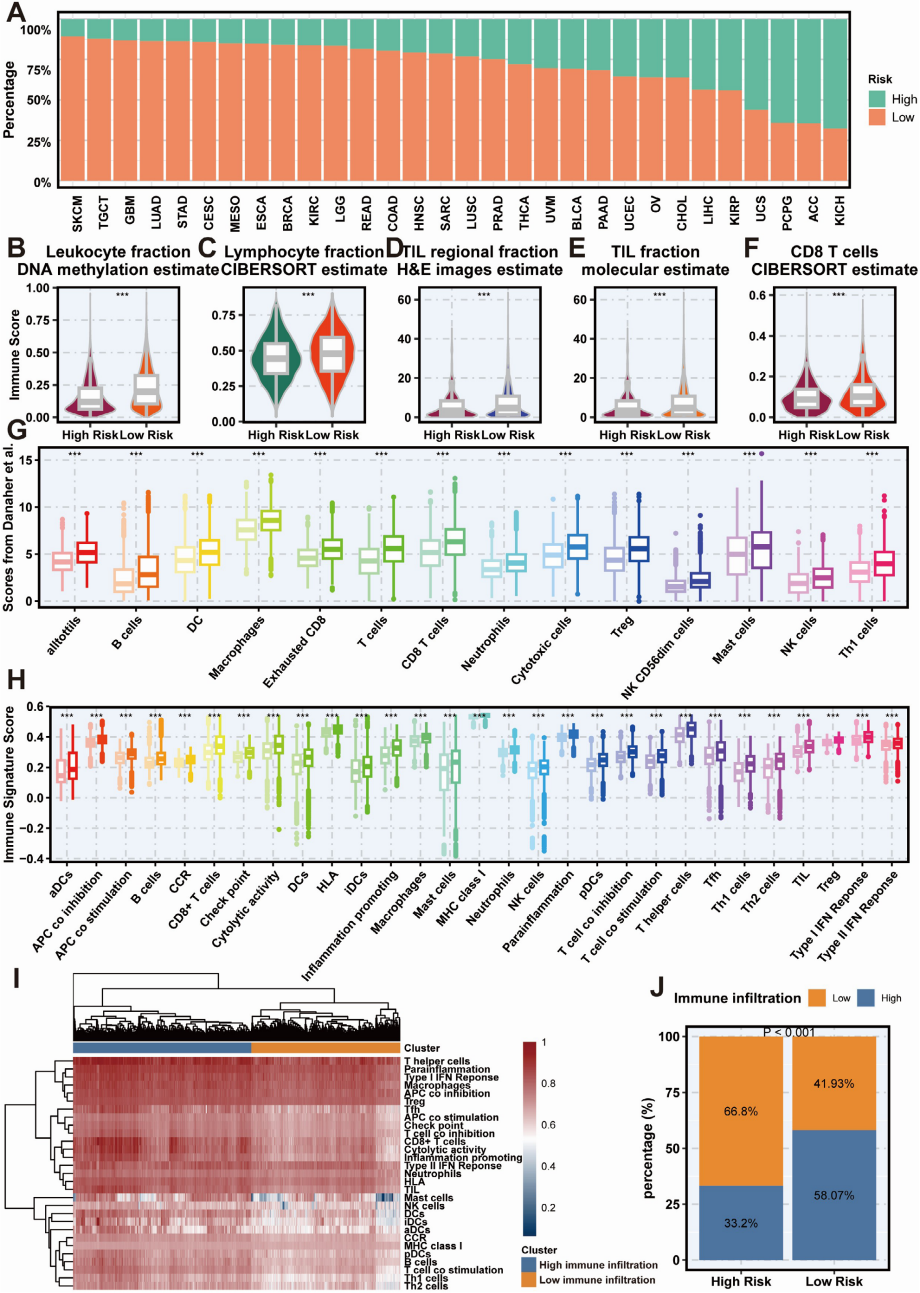
Association between Coagulation Signature (Coagulation.Sig) and immune infiltration across various cancer types. (A) Distribution of high‐risk and low‐risk groups across different cancer types, with percentages represented by bars (high‐risk in red, low‐risk in green). (B–F) Immune infiltration estimates across high‐risk and low‐risk groups: (B) Leukocyte fraction based on DNA methylation, (C) Lymphocyte fraction using CIBERSORT estimates, (D) TIL (tumour‐infiltrating lymphocyte) regional fraction based on H&E image estimates, (E) TIL fraction using molecular estimates, and (F) CD8^+^ T cell fraction using CIBERSORT estimates. Each comparison shows a significant increase in immune cell fractions within the low‐risk group. (G, H) Box plots of immune cell scores and immune signature scores derived from various sources: (G) Immune cell scores based on Danaher et al. including a range of immune cell types (e.g. B cells, macrophages, CD8^+^ T cells), showing higher immune infiltration in the low‐risk group; (H) Immune signature scores, with significant increases in immune activation and cytotoxicity‐related signatures in the low‐risk group. (I) Heatmap showing hierarchical clustering of immune‐related signatures, comparing immune infiltration patterns between high‐risk and low‐risk groups. The low‐risk group predominantly shows high immune infiltration clusters, indicated by the gradient from low (light pink) to high (deep purple) immune infiltration. (J) Proportion of high and low immune infiltration across high‐risk and low‐risk groups. A significantly larger fraction of low‐risk tumours displays high immune infiltration (58.07%) compared to high‐risk tumours (33.2%), supporting the enhanced immune response associated with the low‐risk group (*p* < 0.0001).

**FIGURE 7:**
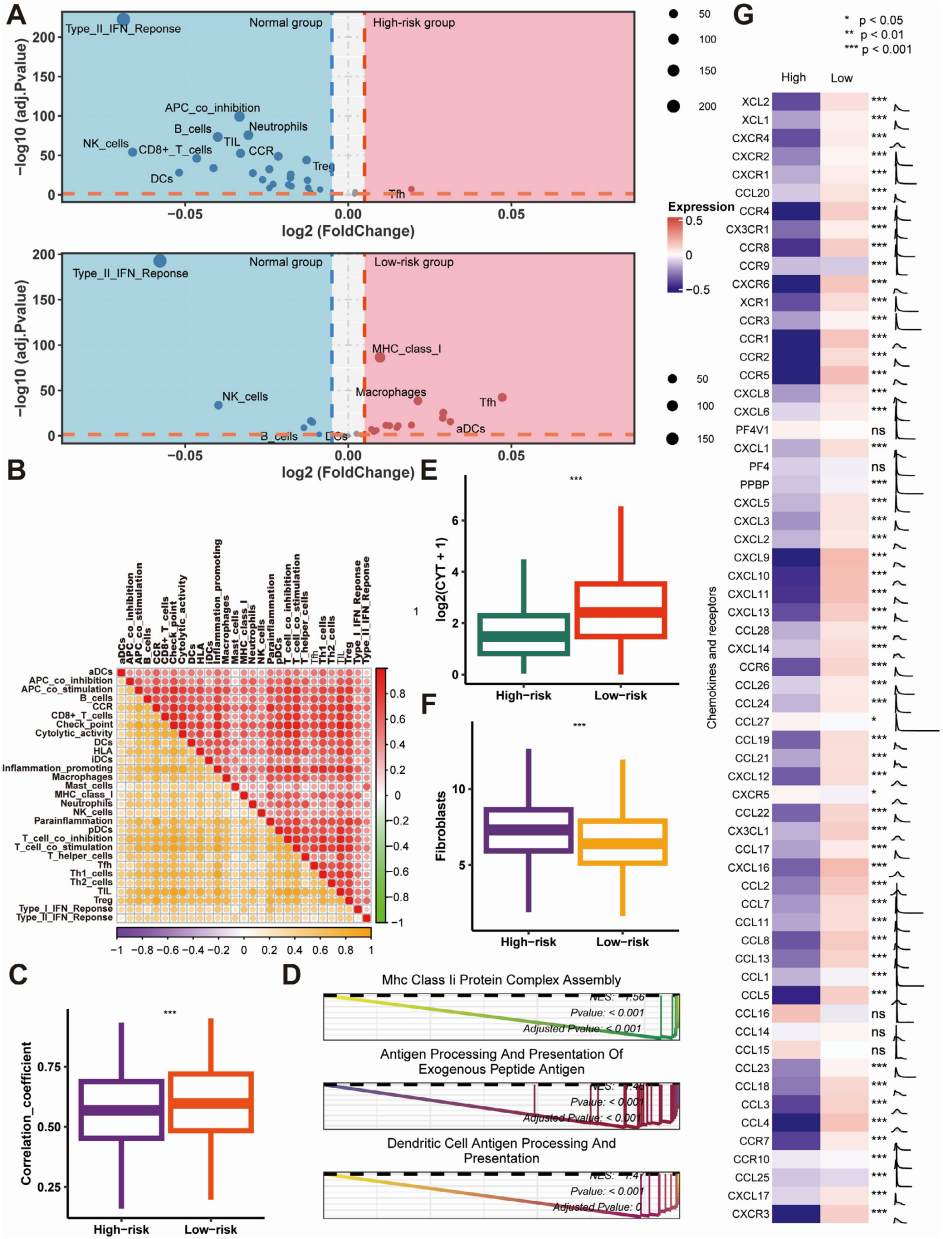
Comparison of immune features, cytokine profiles, and pathway activity between high‐ and low‐risk groups defined by Coagulation Signature (Coagulation.Sig). (A) Volcano plots illustrating differential expression of immune‐related cell types between normal, high‐risk and low‐risk groups. The high‐risk group (top plot) shows enrichment in immunosuppressive cells (e.g. Tregs, Th1), whereas the low‐risk group (bottom plot) shows increased expression of immune‐stimulatory cells (e.g. NK cells, CD8^+^ T cells, DCs) and MHC class I molecules. (B) Correlation matrix of immune signatures, with distinct patterns observed in the high‐risk and low‐risk groups. Positive correlations are shown in red, while negative correlations are shown in blue, indicating complex immune cell interactions within each risk group. (C) Box plots of correlation coefficients comparing immune activities between high‐risk and low‐risk groups, with the low‐risk group showing stronger immune interactions (***p < 0.001). (D) GSEA of pathways related to antigen presentation in the low‐risk group, highlighting significant enrichment in “MHC Class II Protein Complex Assembly,” “Antigen Processing and Presentation of Exogenous Peptide Antigen,” and “Dendritic Cell Antigen Processing and Presentation” pathways, suggesting enhanced antigen presentation capacity in the low‐risk group. (E, F) Box plots comparing cytotoxic activity (E) and fibroblast abundance (F) between high‐risk and low‐risk groups. The low‐risk group exhibits significantly higher cytotoxic activity and lower fibroblast abundance, supporting an immunostimulatory TME in the low‐risk cohort (***p < 0.001). (G) Heatmap showing differential expression of chemokine and receptor genes between high‐risk and low‐risk groups. The low‐risk group demonstrates elevated expression of immune‐stimulatory chemokines, such as CXCL9, CXCL10, and CCL5, which correlate with increased immune cell infiltration and activation. Statistical significance is marked by ***p < 0.001, **p < 0.01, *p < 0.05, and ns = not significant.

A multi‐omics analysis of the TCGA cohort classified patients into high‐ and low‐risk groups based on training cohort criteria (Figure 6A). Low‐risk patients showed higher levels of leukocytes, lymphocytes, and TILs (*p* < 0.001), validated using TIL fractions from Saltz et al.'s method (Figure 6B–E). The low‐risk group had more immune‐stimulatory cells, including CD8^+^ T cells, than the high‐risk group (*p* < 0.001) (Figure 6F). Validation using Danaher et al.'s immune infiltration scores and immune signature scores confirmed more TILs and CD8^+^ T cells in the low‐risk group (*p* < 0.001) (Figure 6G,H). Immune signature clustering validated distinct infiltration patterns, with high immune infiltration predominantly observed in the low‐risk group (*p* < 0.001, Figure 6I,J). The low‐risk group exhibited higher immune signature scores compared to the normal group (Figure 7A). Conversely, no enrichment of immune‐related pathways was observed in the high‐risk group (Figure 7A). Moreover, the correlations among immune activities were significantly stronger in the low‐risk group than in the high‐risk group (Figure 7B,C). GSEA analysis identified 35 significantly enriched pathways in the low‐risk group, including 13 immune‐related pathways such as “Mhc Class II Protein Complex Assembly” (all adjusted *p* < 0.05, Figure 7D). Additionally, low‐risk tumours displayed significantly higher CYT scores (*p* < 0.001) (Figure 7E), while a higher abundance of fibroblasts was detected in the high‐risk group (*p* < 0.001) (Figure 7F). Thus, it is plausible that the chemokine enrichment in low‐risk tumours may boost immune response and improve immunotherapy efficacy.

### 3.6 Differentiating Intrinsic Immunity Between Risk Categories

**FIGURE 8:**
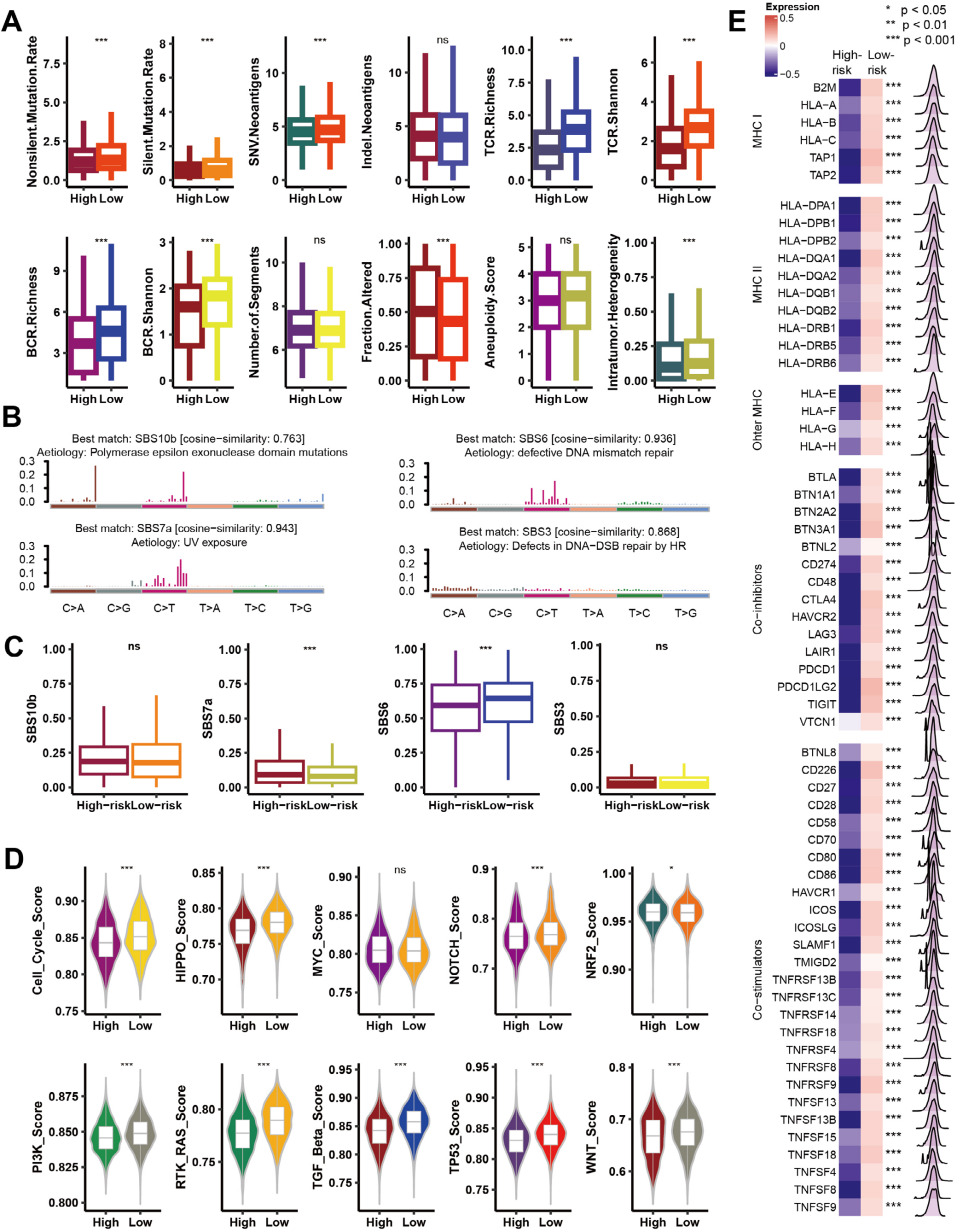
Analysis of mutation rates, immune receptor diversity, genomic instability, and pathway activity between high‐ and low‐risk groups based on Coagulation Signature (Coagulation.Sig). (A) Box plots comparing various genomic and immune metrics between high‐ and low‐risk groups. Metrics include nonsilent mutation rate, silent mutation rate, SNV neoantigens, indel neoantigens, TCR richness and Shannon diversity, BCR richness and Shannon diversity, number of altered segments, fraction altered, aneuploidy score, and intratumor heterogeneity. Significant differences between groups are denoted by **p < 0.01, ***p < 0.001, and “ns” indicates non‐significant results. (B) Mutation signature analysis using cosine similarity to identify mutation signatures associated with different etiologies. Top mutation signatures include SBS10b (polymerase epsilon mutations), SBS7a (UV exposure), SBS6 (defective DNA mismatch repair), and SBS3 (DNA double‐strand break repair defects). (C) Box plots showing the distribution of mutation signature contributions (SBS10b, SBS7a, SBS6, and SBS3) in high‐ and low‐risk groups. Significant differences in mutation signature contributions are observed for SBS7a and SBS6, marked by ***p < 0.001 and ns for non‐significant differences. (D) Heatmap of expression differences in MHC class I/II genes, other MHC molecules, and immune checkpoint inhibitors between high‐ and low‐risk groups, with *p*‐values indicating statistical significance. (E) Violin plots comparing pathway activity scores for various signalling pathways, including cell cycle, HIPPO, MYC, NOTCH, NRF2, PI3K, RTK‐RAS, TGF‐beta, TP53 and WNT, between high‐ and low‐risk groups. Significant differences in pathway activities are noted as ***p < 0.001, **p < 0.01, *p < 0.05, and ns for non‐significant pathways.

Preliminary analysis revealed notable differences in immunogenicity between high‐ and low‐risk groups, with the low‐risk group showing higher mutation frequency and neoantigen load (*p* < 0.05) (Figure 8A). The low‐risk group also showed greater TCR and BCR diversity (*p* < 0.05), while the high‐risk group had more intratumoral heterogeneity (ITH) (*p* < 0.05) (Figure 8A). We identified four mutational signatures from TCGA mutation data: SBS10b, SBS6, SBS7a and SBS3 (Figure 8B). SBS6, linked to DNA mismatch repair and immune response, was more common in the low‐risk group, while SBS7a predominated in the high‐risk group (*p* < 0.05) (Figure 8C) [53]. The low‐risk group had higher enrichment in pathways like cell cycle, Hippo, Notch, PI3K, RAS, TP53, TGF‐beta and WNT (*p* < 0.05) (Figure 8D). In contrast, a greater enrichment of the NRF2 pathway was observed in the high‐risk group (*p* < 0.05), which is linked to immune exclusion [54]. Immune‐related gene expression analysis showed lower MHC class I and II antigen levels in the high‐risk group (*p* < 0.001), indicating immune evasion mechanisms (Figure 8E). The low‐risk group had higher MHC gene expression, suggesting better immunogenicity. Also, immune checkpoint molecules like PD‐1, PD‐L1 and CTLA4 were elevated (*p* < 0.001) (Figure 8E), supporting the potential efficacy of checkpoint inhibitors. These findings highlight the complex relationship between mutations, oncogenic pathways, and immune responses, influencing tumour immunogenicity and treatment outcomes in both risk groups.

### 3.7 BIRC2 Knockdown Inhibits the Malignant Biological Behaviour of A549 and LLC‐1 Cells

The effects of BIRC2 knockdown on A549 and LLC‐1 cells were analysed via gene expression, proliferation, colony formation, and apoptosis. Figure S1A shows that BIRC2 expression levels in A549 and LLC‐1 cells were significantly lower in sh#1 and sh#2 knockdown groups compared to the shNC control, validating the knockdown's effectiveness (Figure S1B,C). Results from the CCK‐8 assay demonstrated that over a 72‐h period, OD450 values in the sh#1 and sh#2 knockdown groups were significantly lower than those in the shNC control group, indicating that BIRC2 knockdown inhibited cell proliferation (Figure S1D,F). The colony formation assay further showed a notable decrease in colony numbers in the sh#1 and sh#2 groups, with quantification confirming that BIRC2 knockdown significantly impaired the clonogenic potential of A549 and LLC‐1 cells (Figure S1G–J). Additionally, apoptosis analysis conducted via flow cytometry, utilising Annexin V and PI staining, revealed that the apoptosis rates in the sh#1 and sh#2 knockdown groups were significantly higher than those in the shNC group. Collectively, these findings suggest that BIRC2 knockdown in A549 and LLC‐1 cells results in decreased gene expression, inhibited proliferation, reduced clonogenic ability, and increased apoptosis, highlighting the critical role of BIRC2 in cell survival and tumour progression.

### 3.8 The Combination of BIRC2 Knockdown and Anti‐PD‐1 Therapy Markedly Reduces Tumour Growth

**FIGURE 9:**
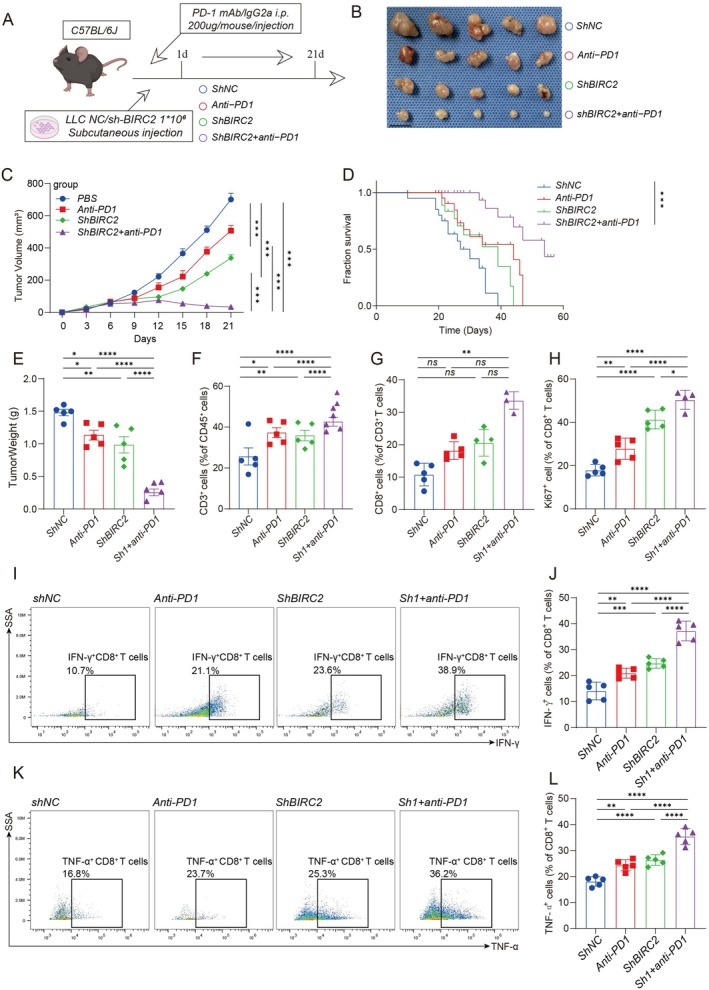
BIRC2 knockdown combined with anti‐PD‐1 therapy significantly inhibits tumour growth. (A) Experimental design schematic: C57BL/6J mice were subcutaneously injected with LLC cells (either shNC or shBIRC2) and treated with either anti‐PD‐1 antibody or control treatment. (B) Representative images of tumours from different treatment groups: ShNC, anti‐PD‐1, shBIRC2 and shBIRC2 + anti‐PD‐1. (C) Tumour growth curves over time for each treatment group. Both shBIRC2 and anti‐PD‐1 alone inhibited tumour growth compared to the control, while the combined treatment group (shBIRC2 + anti‐PD‐1) showed the most significant tumour growth inhibition. (D) Survival curves for each treatment group, with the combined treatment (shBIRC2 + anti‐PD‐1) significantly improving mouse survival. (E) Tumour weight comparison across groups, showing the lowest tumour weight in the combined treatment group, indicating the strongest tumour inhibition effect. (F–H) Proportion of tumour‐infiltrating T cells in different groups, including total CD3^+^ T cells (F), CD8^+^ T cells (G), and proliferating Ki67+ CD8^+^ T cells (H). The combined treatment group showed the highest proportion and activity of CD8^+^ T cells. (I–L) Flow cytometry analysis of IFN‐γ and TNF‐α production in CD8^+^ T cells. Representative flow cytometry plots (I, K) and quantification (J, L) indicate that the combined treatment group had the highest percentage of IFN‐γ + and TNF‐α + CD8^+^ T cells. *Statistical significance: Ns = not significant, *p < 0.05, **p < 0.01, ***p < 0.001, ****p < 0.0001.

Among the CoagulationSig modelling genes, BIRC2 was identified as a potential therapeutic target and subsequently validated through further experiments. As shown in Figure 9A, the experimental design included four treatment groups: ShNC, anti‐PD‐1 monoclonal antibody (Anti‐PD1), BIRC2 knockdown (ShBIRC2), and BIRC2 knockdown combined with anti‐PD‐1 (ShBIRC2 + anti‐PD1). C57BL/6J mice were subcutaneously injected with either LLC (Lewis Lung Carcinoma) control or sh‐BIRC2 cells. Anti‐PD‐1 (200 μg/mouse) was administered every 3 days starting from day 0, for a total of seven doses. The results indicated that BIRC2 knockdown in conjunction with anti‐PD‐1 treatment significantly inhibited tumour growth and reduced tumour volume (Figure 9B,C), and prolonged mouse survival (Figure 9D). Analysis of immune cell populations (Figure 9E–H) showed a marked increase in CD3^+^ T cells, CD8^+^ T cells, and Ki67+ proliferating CD8^+^ T cells in the combination treatment group. Flow cytometry (Figure 9I–L) revealed higher IFN‐γ and TNF‐α levels in CD8^+^ T cells from the combination group. Collectively, these results demonstrate that combining BIRC2 knockdown with anti‐PD‐1 therapy boosts anti‐tumour immunity, making BIRC2 a potential therapeutic target.

## 4 Discussion

The tumour coagulome fosters an inflammatory TME, recruiting and activating infiltrating cells like platelets, neutrophils, polymorphonuclear cells, macrophages and monocytes. This process leads to a series of thrombotic events known as immunothrombosis. In cases of systemic infections, these immunothrombotic responses can cause microvascular blockages, resulting in multiple organ dysfunction due to compromised blood flow and nutrient delivery [55]. Conversely, tumours exploit these immunothrombotic responses to evade apoptosis and escape immunosurveillance. The investigation into the functions of the tumour coagulome has gained significant attention regarding its implications for the TME. In this context, we conducted an innovative study to explore the potential role of the tumour coagulome in enhancing the efficacy of ICI treatments across various cancer types. Additionally, our research aimed to deepen the understanding of the molecular mechanisms associated with the tumour coagulome by employing a multi‐omics approach. This multi‐omics strategy allows for a comprehensive analysis of the interactions between coagulation factors and immune responses within the TME, potentially unveiling new therapeutic avenues. By integrating various omics data, we can better understand how the tumour coagulome influences immune modulation and contributes to treatment outcomes in cancer therapy.

This is the first study to explore the link between the tumour coagulome and ICI therapy outcomes across pan‐cancer. We developed a novel Coagulation.Sig signature, termed Coagulation.Sig, through an integrative analysis of RNA‐Seq datasets across various cancer types. This signature aims to predict responses to ICIs, a class of drugs that have transformed cancer treatment but often exhibit limited effectiveness due to the absence of robust predictive biomarkers [56, 57].

The study's methodology encompasses a comprehensive analysis of large‐scale pan‐cancer data, including TCGA cohorts and multiple ICI transcriptomic cohorts. This extensive approach facilitates a more generalised and robust validation of the Coagulation.Sig's predictive capabilities across different cancers. Furthermore, we demonstrated that the predictive performance of Coagulation.Sig surpasses previously established signatures, achieving an AUC greater than 0.7 in validation, testing and in‐house sets, indicating high predictive accuracy. This study explores intrinsic and extrinsic immune landscapes in the TME. TCGA multi‐omics analysis revealed differences in immune infiltration between high‐ and low‐risk groups, providing insights into tumour‐immune interactions. Low‐risk tumours showed higher immune cell infiltration, with elevated CD8^+^ T cells and immunostimulatory cells, indicating greater tumour immunogenicity. These findings highlight the robustness and broad applicability of the Coagulation.Sig.

Six machine learning models were used to develop Coagulation.Sig, a reliable biomarker for predicting ICI responses and identifying patients who may gain survival benefits. Coagulation.Sig was compared with six pan‐cancer and seven melanoma‐specific leading signatures. Coagulation.Sig exhibited superior generalisation capabilities compared to pan‐cancer signatures and maintained robust performance across diverse cohorts. When compared to melanoma‐specific signatures, Coagulation.Sig consistently ranked among the top three, achieving a competitive AUC of 0.72. In practical application, Coagulation.Sig helps to prevent unnecessary immune‐related adverse events in patients unlikely to derive significant benefits from treatment. Furthermore, given the substantial cost of treatment, often exceeding $120,000 [58], implementing biomarker‐driven approaches that enhance diagnostic accuracy can mitigate considerable financial burdens associated with treatments predicted to yield minimal improvements. As RNA sequencing technology advances and becomes more accessible, clinical laboratories are increasingly equipped to efficiently and affordably monitor comprehensive gene expression profiles, facilitating the computation of the Coagulation.Sig score. Overall, Coagulation.Sig can serve as a critical biomarker for identifying patients most likely to benefit from ICI therapies. By integrating this signature into routine clinical assessments, oncologists can make more informed decisions regarding treatment plans, potentially improving patient outcomes while minimising unnecessary exposure to ineffective therapies.

This study has several limitations. First, it uses data from 10 RNA‐Seq cohorts spanning six cancer types. While the inverse correlation between Coagulation.Sig and anti‐tumour immunity across 30 cancer types is promising, pan‐cancer validation requires future ICI trials. Second, missing clinical data, including sex, age, tumour stage, and TMB, limit multivariate regression analysis and complicate biomarker assessment.

## 5 Conclusions

In this research, we established the Coagulation.Sig signature, which has significant potential for personalising treatment strategies for patients across various cancer types. This signature will enable the development of innovative approaches to clinical decision‐making and management. Additionally, our study enhances understanding of the molecular mechanisms underlying the TME through a multi‐omics approach.

## Author Contributions

**Ziang Yao:** data curation (equal), software (equal), visualization (equal), writing – original draft (equal). **Jun Fan:** formal analysis (equal), visualization (equal), writing – original draft (equal). **Yucheng Bai:** conceptualization (equal), data curation (equal), software (equal). **Jiakai He:** conceptualization (equal), data curation (equal), software (equal), supervision (equal). **Xiang Zhang:** conceptualization (equal), data curation (equal), visualization (equal), writing – original draft (equal). **Renquan Zhang:** methodology (equal), project administration (equal), writing – original draft (equal), writing – review and editing (equal). **Lei Xue:** data curation (equal), formal analysis (equal), investigation (equal), writing – original draft (equal), writing – review and editing (equal).

## Ethics Statement

The animal experiment was approved by the Animal Experiment Ethics Committee of the First Affiliated Hospital of Nanjing Medical University and was conducted in accordance with animal experiment standards.

## Conflicts of Interest

The authors declare no conflicts of interest.

## Supporting information


**Figure S1:** BIRC2 knockdown inhibits the malignant biological behaviours of A549 and LLC‐1 cells. (A) BIRC2 expression levels in A549 and LLC‐1 cell lines were assessed by quantitative PCR following transfection with shNC (negative control), sh#1, or sh#2. Both sh#1 and sh#2 significantly reduced BIRC2 expression levels compared to shNC in both cell lines. Statistical significance: ****p < 0.0001. (B, C) Cell proliferation assays for A549 (B) and LLC‐1 (C) cells transfected with shNC, sh#1, or sh#2 were performed over a period of 72 h (A549) and 96 h (LLC‐1), respectively. Both BIRC2 knockdowns (sh#1 and sh#2) showed reduced proliferation compared to the shNC control. (D) Representative images of colony formation assays for A549 and LLC‐1 cells following BIRC2 knockdown. Cells transfected with sh#1 and sh#2 formed fewer colonies than those transfected with shNC. (E, F) Quantification of colony numbers for A549 (E) and LLC‐1 (F) cells, showing significant reductions in colony formation in BIRC2 knockdown groups compared to shNC. Statistical significance: *p < 0.05, **p < 0.01, ***p < 0.001. (G–J) Flow cytometry analysis of apoptosis in A549 (G, H) and LLC‐1 (I, J) cells. Representative scatter plots (G, I) and quantification of apoptosis percentages (H, J) indicate increased apoptosis rates in BIRC2 knockdown groups (sh#1 and sh#2) compared to shNC. Statistical significance: **p < 0.01, ****p < 0.0001.

## Data Availability

All datasets pertinent to this study are accessible through the TCGA database (http://cancergenome.nih.gov/) and GEO database (https://www.ncbi.nlm.nih.gov/geo/). For further inquiries or data requests, interested parties are advised to reach out to the corresponding authors.
