# Supplementary figures and images for: Unravelling Cancer Immunity: Coagulation.Sig and BIRC2 as Predictive Immunotherapeutic Architects
Source: J Cell Mol Med. 2025 Mar 30;29(7):e70525. doi: 10.1111/jcmm.70525 (PMC11955421; doi:10.1111/jcmm.70525)

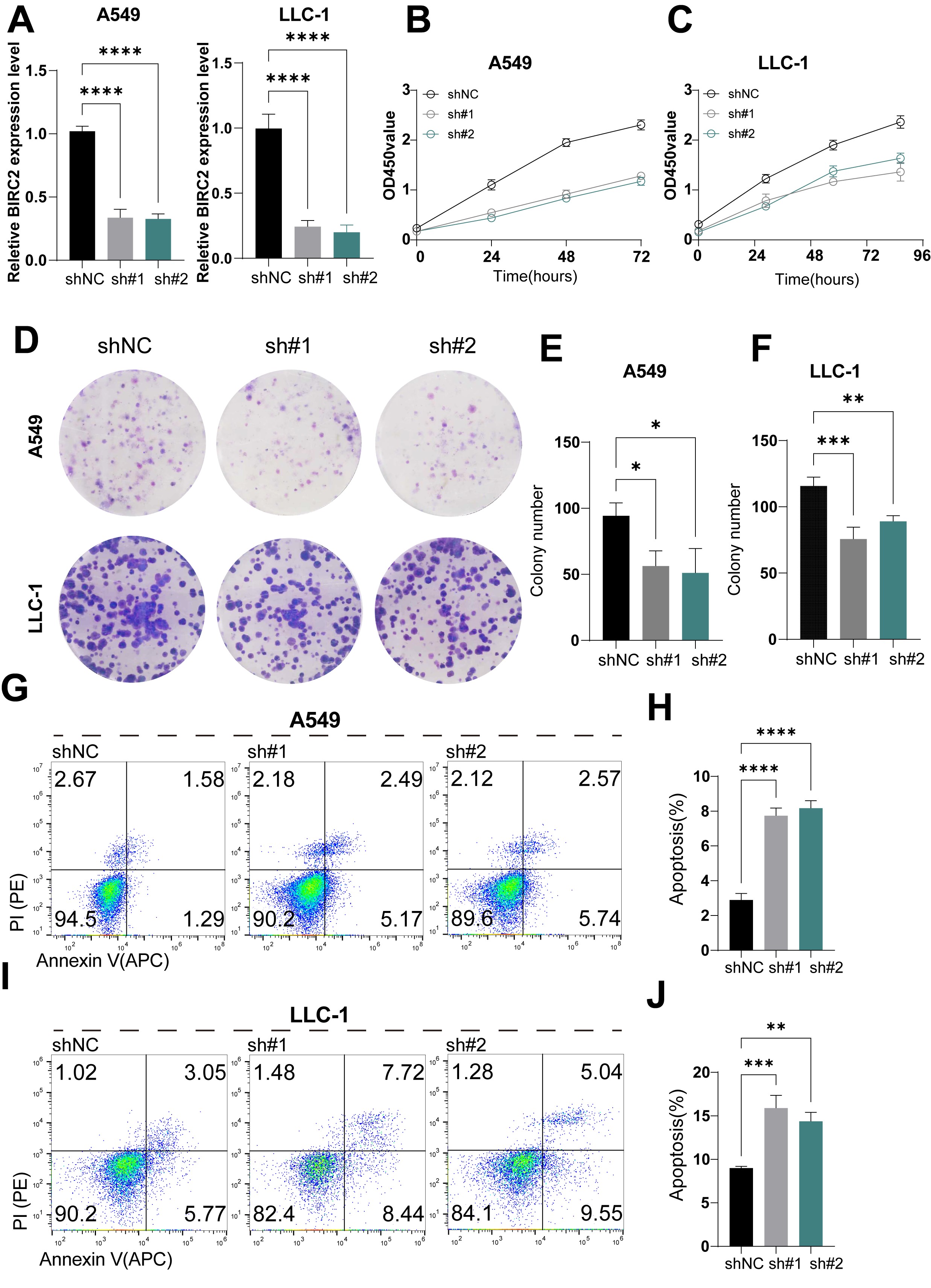

Supplement: Supplementary file 1 — Figure S1: BIRC2 knockdown inhibits the malignant biological behaviours of A549 and LLC‐1 cells. (A) BIRC2 expression levels in A549 and LLC‐1 cell lines were assessed by quantitative PCR following transfection with shNC (negative control), sh#1, or sh#2. Both sh#1 and sh#2 significantly reduced BIRC2 expression levels compared to shNC in both cell lines. Statistical significance: ****p < 0.0001. (B, C) Cell proliferation assays for A549 (B) and LLC‐1 (C) cells transfected with shNC, sh#1, or sh#2 were performed over a period of 72 h (A549) and 96 h (LLC‐1), respectively. Both BIRC2 knockdowns (sh#1 and sh#2) showed reduced proliferation compared to the shNC control. (D) Representative images of colony formation assays for A549 and LLC‐1 cells following BIRC2 knockdown. Cells transfected with sh#1 and sh#2 formed fewer colonies than those transfected with shNC. (E, F) Quantification of colony numbers for A549 (E) and LLC‐1 (F) cells, showing significant reductions in colony formation in BIRC2 knockdown groups compared to shNC. Statistical significance: *p < 0.05, **p < 0.01, ***p < 0.001. (G–J) Flow cytometry analysis of apoptosis in A549 (G, H) and LLC‐1 (I, J) cells. Representative scatter plots (G, I) and quantification of apoptosis percentages (H, J) indicate increased apoptosis rates in BIRC2 knockdown groups (sh#1 and sh#2) compared to shNC. Statistical significance: **p < 0.01, ****p < 0.0001. [file JCMM-29-e70525-s001.jpg]
